# The role of apheresis and insulin therapy in hypertriglyceridemic acute pancreatitis—a concise review

**DOI:** 10.1186/s12876-023-02957-3

**Published:** 2023-10-03

**Authors:** Jakob Gubensek

**Affiliations:** 1 Department of Nephrology, University Medical Center Ljubljana, Zaloska cesta 7, 1000 Ljubljana, Slovenia; 2 Faculty of Medicine, University of Ljubljana, Ljubljana, Slovenia

**Keywords:** Therapeutic plasma exchange, Double-filtration plasmapheresis, Hyperlipidemic pancreatitis, Hypertriglyceridemia, Free fatty acids, Conservative treatment, Pregnancy

## Abstract

Severe hypertriglyceridemia (HTG) is the third most common cause of acute pancreatitis (AP) and is involved in its pathogenesis. Chylomicrons increase blood viscosity and induce ischemia, while free fatty acids induce inflammation and distant organ damage. Conservative treatment options include fasting and insulin; limited evidence shows their comparable efficacy. Plasma exchange might provide more rapid lowering of triglycerides and amelioration of systemic effects of severe AP. Available data from controlled studies show only moderately faster lowering of triglycerides with apheresis (about 70% vs. 50% with conservative treatment within 24 h) and limited data from non-randomized studies show no improvement in clinical outcomes. New evidence is expected soon from ongoing large randomized trials. Until then, insulin may be used in mild HTG-AP and plasma exchange should be considered only in severe HTG-AP, especially if the decline of triglycerides with conservative treatment is slow, and in HTG-AP during pregnancy.

## Introduction

The association between hypertriglyceridemia (HTG) and acute pancreatitis (AP) was first described by Speck as early as 1865 [1]. HTG is often considered the third most common cause of AP [2] and accounts for about 2–10% of cases in the general population [2–6] and up to 28–48% of cases during pregnancy [7, 8], where it is the second most common cause of AP [9]. The overall mortality of AP is low, about 2%, but increases to 15–20% in severe pancreatitis, defined by organ failure persisting beyond 48 h [2]. The reduction of TG with insulin in HTG-AP was first described by Bagdade in 1967 in a patient with deranged diabetes [10]. A decade later, in 1978, Betterige described the first use of therapeutic plasma exchange (TPE) and noted that it was able to reduce TG much faster than insulin [11]. The use of apheresis, either TPE or any form of lipoprotein apheresis, has since often been used in clinical practice to treat HTG-AP, especially in centers with good availability of apheresis, although the evidence for this treatment is still poor and many centers never use it. The use of insulin is often advocated as simple and safe, but also lacks firm evidence. This review aims to summarize current knowledge on the role of both treatments for HTG-AP with focus on the possible mechanisms by which TPE could ameliorate the course of HTG-AP.

## Pathogenesis of hypertriglyceridemic acute pancreatitis

It is generally believed that triglycerides above 10 mmol/L (equal to 882 mg/dL, but a round value of 1000 mg/dL is usually used in the literature) should be considered a causative factor for AP, although it is well known that very high triglycerides can occur without AP. The 2012 Endocrine Society guidelines only consider very severe HTG (above 2000 mg/dL) as a risk factor for development of pancreatitis [12]. In 2016, a large population study nicely showed that the risk of developing AP actually starts increasing at (non-fasting) triglyceride levels above 2 mmol/L (177 mg/dL) [13]. Probably, there is a level at which the future risk of AP starts increasing (as one can expect higher peaks in the future in such a patient due to a metabolic disorder) and another level at which triglycerides actually trigger an episode of AP. From a diagnostic point of view, it should be acknowledged, that triglyceride levels can drop fast with fasting, so the timing of triglyceride measurement in relation to the onset of AP is important.

The pathophysiological mechanisms that trigger an episode of AP in very severe hypertriglyceridemia are not yet fully understood. One of likely mechanisms is formation of chylomicrons, very large lipoprotein particles, in the blood, when triglyceride levels reach 10–20 mmol/L. This results in grossly lipemic serum and increased blood viscosity, inducing and maintaining ischemia in the pancreatic capillary bed [14]. The other likely mechanism is increased formation of free fatty acids (FFA) resulting from hydrolysis of excess triglycerides by pancreatic lipase [14, 15]. When they exceed binding capacity of albumin, FFAs are toxic to the acinar cells and induce inflammation within the pancreas, which increases cytokine levels [15]. FFAs also damage endothelial cells and induce distant organ damage, especially acute lung injury [15–18]. Furthermore, lipotoxicity may also result from hydrolysis of the adipose tissue surrounding the pancreas in obese patients, resulting in more severe course of AP in obese patients, regardless of AP etiology [19, 20].

Given involvement of triglycerides and lipotoxicity in aggravation of AP, one would expect severity of hypertriglyceridemia to be related to severity of AP and frequency of systemic complications. Some studies have shown this [6, 21–23], but in other large cohorts, triglycerides were not associated with disease severity or mortality [24, 25]. Nevertheless, given their pathogenic role and multiple mechanisms leading to distant organ damage, rapid lowering of triglycerides in HTG-AP is generally considered to be an important treatment goal.

## Summary of current guidelines

There are quite some guidelines, pertinent to HTG-AP, with some heterogeneity among their recommendations. The 2012 Endocrine Society guideline on the evaluation and treatment of HTG does not recommend the use of plasmapheresis in the treatment of very severe HTG with AP [12]. The 2020 European Atherosclerosis Society task force consensus statement on rare dyslipidemias also does not recommend the use of TPE in chylomicronemia syndrome with AP, with the possible exception of controlling severe HTG due to monogenic chylomicronemia during pregnancy; it only recommends fasting and intravenous insulin for patients with diabetes [26].

Gastroenterology guidelines for the management of AP usually only mention HTG as a cause of AP and make no reference to apheresis treatment at all [27–29], with the exception of the guidelines issued by the French Society of Anesthesia and Intensive Care Medicine and the French National Society of Gastroenterology in 2021, which do suggest initiation of TPE to rapidly reduce severe HTG in patients admitted to intensive care for severe HTG-AP in the event of medical treatment failure [30].

Finally, there are two guidelines from the field of apheresis medicine. The 2023 American society of apheresis guidelines [31] consider severe HTG-AP as a category 3 indication and the 2021 Japanese apheresis guidelines have the same categorization for HTG-AP, without mentioning of the disease severity [32]. The category 3 means that the optimal role of apheresis is not established and individualized decision making is paramount, but also that further evidence is necessary.

## Conservative treatment with insulin

Conservative treatment of HTG-AP includes fasting, fluid replacement and analgesics. To specifically target triglyceride (and FFA) metabolism, heparin and insulin have been used. Heparin releases endothelial lipase from the endothelium, which transiently increases circulating lipoprotein lipase levels and triglyceride metabolism, but later actually causes lipoprotein lipase deficiency due to increased hepatic degradation [33, 34]. Therefore, use of heparin is not recommended any more for this purpose [12, 34]. Insulin increases the activity of lipoprotein lipase in adipose tissue [34, 35]. After initial description by Bagdade [10], its use was described in patients with elevated glucose levels [36] or even overt diabetic ketoacidosis, but also in patients without hyperglycemia or diabetes in whom insulin was administered concomitantly with glucose [37]. Although there are quite some reports of conservative treatment of HTG-AP, which often includes insulin, the insulin dose is rarely reported and almost never prescribed in a protocolized manner. Quite different dosing schemes are described in the literature. A low dose of insulin can be given to cover the metabolic needs of concomitant glucose infusion. This usually means 4–6 IU of insulin added to 500 ml of 5% glucose (or 5% glucose in normal saline, which is better for volume replacement) and is sometimes referred to as a 1:4 or 1:6 insulin to glucose ratio (i.e., 4–6 IU of insulin per 25 g of glucose contained in 500 ml). The rate of this glucose infusion with added insulin is often not specified, but an infusion rate of e.g. 250 ml/h would result in giving insulin at approx. 2–3 IU/h [38, 39]. High-dose insulin therapy, on the other hand, is the dose usually used in diabetic ketoacidosis or hyperglycemic hyperosmolar nonketotic syndrome, i.e. 0.1–0.3 IU/kg body weight/h intravenously, usually given as pure insulin separately by perfusor, which corresponds to about 7–20 IU/h in an average-sized patient [38]. This high dose also requires glucose substitution sooner or later, usually given separately, and also requires intensive blood glucose monitoring. Subcutaneous administration of insulin has also been used in some cases [37], but is likely less effective.

Reported lowering of triglycerides in cohorts using intravenous insulin was 40–68% within the first 24 h [38, 40, 41]. As decline in triglycerides after the initiation of fasting and other therapies is exponential and thus dependent on the baseline values, expressing it as % of baseline value seems appropriate. A comparison of non-intensive and intensive insulin therapy showed similar therapeutic efficacy and also a similar incidence of hypoglycemic events [38]. Therefore, low-dose insulin seems to be easier to use, unless there is severe hyperglycemia present. There is only one non-randomized study comparing insulin therapy with fasting-only, which did not show a more rapid decline in triglycerides with insulin [42]. Therefore, there is currently no evidence that insulin is more effective than fasting alone [43].

## Potential beneficial effects of TPE

### Rapid lowering of triglycerides

The speed of lowering the triglycerides was the initial advantage of TPE observed by Betterige in 1978 [11]. The sight of milky plasma being removed from the patient’s circulation [44] requires almost no additional convincing of TPE efficacy, but as it has turned out over the last few years, the story is not so simple. Timely reduction of serum triglycerides in the early phase of HTG-AP was associated with decreased development of persistent organ failure [23], which is the hallmark of severe AP. Triglycerides and chylomicrons are usually cleared from the circulation within a few hours after a meal in normal-weight individuals, but their metabolism is impaired in obese [45, 46] and diabetic patients [47]. Furthermore, the time to reach a “safe” triglycerides level, often considered to be below 10 mmol/L, depends on the initial levels.

Comparative data on triglyceride reduction with apheresis is accumulating in the literature. In a retrospective cohort study, we showed a much greater reduction in triglycerides during 24-hour periods with TPE as compared to 24-hour periods during which no TPE was performed (59% vs. 27%, p < 0.001) [24], but this comparison is problematic, since the periods without TPE were often late in the course of disease and decrease in triglycerides is exponential. Later, two small retrospective studies showed a similar time-course of triglycerides whether TPE was performed or not [48, 49]. It could be argued that the decrease in the first 24 h is a better outcome measure than a time-course over several days for what could be considered as an “emergency” treatment. Additionally, retrospective comparisons are commonly affected by confounding by indication. Furthermore, after a handful of case reports of conservative treatment, a large cohort of patients treated without TPE was finally published showing a median reduction in triglycerides of 44% within the first 24 h, which is a very good result compared with apheresis treatment [25]. There were two further large retrospective cohort studies from China comparing apheresis with conservative treatment. In one study 80% reduction was achieved within first 24 h with double-filtration plasmapheresis (DFPP) as compared to 68% with conservative treatment [50]. In the other 71% vs. 48% reduction was observed with TPE [51]. A meta-analysis of 15 observational controlled studies comparing different apheresis techniques with conventional treatment was just published and showed that apheresis achieved a significantly higher reduction rate of triglycerides within 24 h [52]. Finally, trying to settle this issue, two randomized studies were published recently. A large RCT from China reported a significantly higher median triglyceride reduction of 68% within the first 24 h with TPE vs. only 52% with conservative treatment (which was not specified) [53]. Similarly, our small RCT showed a borderline greater 67% reduction vs. 53% with insulin therapy [39].

From these relatively consistent data accumulated over the past years, it appears that apheresis has some, but relatively small (approx. 70% vs. 50% reduction within 24 h), advantage over conservative treatment, which often includes insulin. This difference seems of questionable clinical significance, especially if the invasiveness, cost and lack of availability of apheresis treatment in some centers is taken into the account. However, this small difference could become significant at extremely high baseline triglycerides, e.g., above 50 mmol/L [54], as it could affect the time in which triglycerides are lowered below 10 mmol/L.

In addition to the decrease in triglycerides, i.e. removing the causative factor for HTG-AP, TPE also reduces blood viscosity, improves blood rheology and likely improves perfusion in capillary beds, similar to what is achieved by fibrinogen and other large molecules removal in so-called rheopheresis, e.g. in microvascular disease.

### Removal of proteases and replacement of lipase and protease inhibitors

It has been postulated that removal of pancreatic proteases and replacement of protease inhibitors (mainly α2 macroglobulin) might be an important mechanism for the efficacy of TPE [34, 55]. If fresh frozen plasma (FFP) is used as a replacement solution during TPE, this can replace lipoprotein lipase and protease inhibitors. Antiproteases have an important role in binding and eliminating pancreatic enzymes released from damaged pancreatic tissue into the bloodstream. Although a RCT with high-volume FFP infusion in AP found increased plasma concentrations of several naturally occurring antiproteases (e.g. α2 macroglobulin) during AP, there was no improvement in clinical outcome [55]. Although the use of FFP replacement during TPE for HTG-AP is widespread in China, without proven benefit and with a higher possibility of allergic reactions, its use does not seem warranted.

### Amelioration of systemic effects of severe acute pancreatitis

TPE with FFP has been used in patients with septic shock with variable success, making it a category 3 indication in the ASFA guidelines [31]. A small pilot RCT demonstrated that a single TPE with FFP improves hemodynamic status and vasopressor requirement [56] and corrects factors involved in platelet activation and hemostasis (such as ADAMTS13, antithrombin III, protein C, von Willebrand factor) [57]. A retrospective propensity score matched analysis comparing septic patients who did or did not receive TPE showed no effect on mortality and even a lower number of ICU-free-and-alive days in the TPE group, with some evidence of residual imbalances between groups [58]. A larger RCT showed a significant improvement in APACHE III score in the TPE group and significantly lower 28-day mortality (p = 0.05), which became borderline significant (p = 0.07) after adjustment for baseline imbalances, including a significant age difference [59]. Therefore, the evidence for treatment of septic shock with TPE is not yet fully convincing. Like sepsis, severe AP triggers a systemic inflammatory response syndrome (SIRS). Therefore, TPE may have similar beneficial effects in severe HTG-AP with SIRS by removing inflammatory mediators and restoring coagulation and complement regulation. Furthermore, during inflammation CRP molecule itself is involved in marking damaged cells for phagocytosis, a process which can also induce damage to still viable cells, therefore increasing damage to the tissues induced by hyperinflammation [60]. It was shown that CRP is removed and lowered by TPE [61], but the clinical significance of this in general and in the context of AP is uncertain. There are some promising results in the literature with selective CRP adsorption in other acute states (acute myocardial infarction [62]) and a controlled study is ongoing also in patients with AP (DRKS00014265).

As discussed previously, FFAs induce distant organ damage in HTG-AP and are associated with increased mortality. FFAs also inhibit lipoprotein lipase and therefore the degradation of triglyceride-rich lipoproteins, more so in patients with diabetes [47]. Given the low molecular weight of FFAs (approx. 150–300 Da), they are removed by TPE, which might be beneficial, but they are not removed by DFPP, as they are filtered back by the secondary filter [63]. Data on FFA in HTG-AP are very scarce in the literature. In our small RCT, no difference was found between the FFA levels in the TPE group as compared to the insulin group [39]. In the ongoing ELEFANT trial, although the elimination of FFAs is cited as the rationale for the study, measurement of FFA levels is not among secondary outcomes [64], but hopefully this will be measured as a post-hoc analysis. It should be noted that insulin also lowers circulating FFA levels, more so in non-obese patients [65]. The main mechanism for this is the suppression of intracellular hormone-sensitive lipase, which reduces endogenous release of FFA from fat tissue [66].

### Improvement in clinical course

All the described potential beneficial mechanisms of TPE in HTG-AP are only an intermediate or surogate treatment goal. The main goal is improvement in clinical course of AP, reduction in local and systemic complications and ultimately reduction of mortality. Unfortunately, the evidence that this can be achieved with TPE treatment is still lacking.

The first comparison of TPE with conservative treatment in a small cohort of HTG-AP patients was performed almost 20 years ago and found no effect on the incidence of local or systemic complications or mortality [67]. Recently, Lu published a large propensity score matched retrospective comparison of DFPP and conservative management in HTG-AP [50]. Although their comparison showed rapid and efficient triglyceride lowering with DFPP, there was no beneficial effect on clinical outcomes of AP, including persistent organ failure, local complications, length of stay and in-hospital mortality, not even in the severe AP group [51]. The observed higher incidence of respiratory failure in the TPE group was likely the result of residual confounding by indication rather than a complication of TPE [50, 63]. Hutchison et al. published a large cohort of 115 HTG-AP episodes treated conservatively without TPE (but with insulin in 47% of cases) [25]. Compared to published cohorts, treated with TPE, there was no significant difference in mortality and there was also no difference in the rate of local complications or need for mechanical ventilation [25]. A recent meta-analysis of observational studies also found no improvement in local or systemic complications with apheresis treatment; while mortality was in fact higher, a trial sequential analysis showed that this was likely a false positive finding [52]. In our small randomized trial, which was underpowered for such a comparison, there was a similar number of patients with severe course of AP and similar maximum CRP levels in the TPE and insulin groups; all patients survived [39].

### Hypertriglyceridemic pancreatitis during pregnancy

AP during pregnancy is a rare condition that occurs approximately once in 1,000–10,000 pregnancies and often occurs in the third trimester [9, 68, 69]. Hormonal changes during pregnancy lead to an increase in triglycerides, especially in patients with an underlying disorder of lipid metabolism [68, 69], making hypertriglyceridemia the second most common cause of AP during pregnancy [9]. An episode of AP in pregnant women not only endangers the life of the mother, but also increases fetal mortality, which is about 11%, but can be as high as 44% in severe AP [9], as AP of any etiology often requires delivery or termination of pregnancy. The use of medications in limited during pregnancy [69] and TPE should be considered, possibly as a preventive measure in severe hypertriglyceridemia without AP, but especially in the case of HTG-AP [69], although reports are limited. TPE is well tolerated during pregnancy and complications are rare [70, 71].

### Upcoming randomized trials

There are several randomized controlled trials registered in the WHO clinical trials registry, which plan to recruit patients with (severe) HTG-AP and aim to compare TPE with conservative therapy / insulin or insulin with conservative therapy (see Table 1). The vast majority of registered trials are from China, where apheresis is widely used. It is possible that not all of them are actually active or ever started, as some should have already been completed by now, but they may have been delayed due to the COVID-19 epidemic and their status has not been updated. I believe that the one, which will provide the most definitive data, is the ELEPHANT trial (ISRCTN41530928), which is the biggest, with almost 500 patients planned. Importantly, this trial has TPE, insulin and conservative arms, so it should provide data on both therapies, possibly settling the question of optimal HTG-AP therapy in the near future [64].

**Table 1:** Summary of registered randomized trials on the role of therapeutic plasma exchange or insulin therapy in the setting of hypertriglyceridemic acute pancreatitis from the clinical trials registry

Trial No, acronym	study design, main country	N of patients (planned)	inclusion criteria	treatment arms	status of the study
NCT03342807, Bi-TPAI trial [72]	multicentric RCT, China	220	HTG-AP	1) intensive insulin (0.1–0.3 IU/kg/h) ± glucose 2) daily TPE	planned 2017–2020, protocol published, status unclear
NCT03501680	RCT, China	200	moderate / severe HTG-AP	1) intensive insulin (glucose 4.5-6 mmol/l) 2) standard insulin (glucose 8–10 mmol/l) 3) TPE	planned 2018–2020
ChiCTR1800020415	multicentric RCT, China	190	severe HTG-AP	1) TPE 2) conservative	planned 2019–2021, recruiting
ChiCTR2000037754	single-center RCT, China	60	HTG-AP	1) DFPP 2) conservative	planned 2020–2022
ChiCTR1900022028	multicentric RCT, China	178	HTG-AP	1) TPE 2) CRRT	planned 2019–2022
ChiCTR2100049081	single-center RCT, China	180	HTG-AP + SIRS	1) TPE 2) conservative	planned 2021–2023
NCT05487833	single-center RCT, Slovenia	30	HTG-AP	1) low dose insulin (4 IU / 500 ml 5% glucose in normal saline) 2) conservative	planned 2022–2024
ISRCTN41530928, ELEFANT trial [64]	multicentric, Hungary	495	HTG-AP	1) TPE 2) intensive insulin (0.1 IU/kg/h) + 5% glucose + enoxaparin 4000 IU / 12 h s.c. 3) fluids only	planned 2020–2024

CRRT—continuous renal replacement therapy, HTG-AP—Hypertriglyceridemic acute pancreatitis, RCT—randomized controlled trial, SIRS—systemic inflammatory response syndrome, TPE—therapeutic plasma exchange

## Conclusions and suggestions for clinical practice

In conclusion, existing data from randomized and non-randomized controlled studies are quite consistent, showing only a moderately faster lowering of triglycerides with TPE within the first 24 h (about 70% as compared to about 50% with conservative therapy including insulin) [39, 51, 53]. By itself, this small difference is unlikely to be clinically significant in the majority of patients. Furthermore, there are no definitive observational data on beneficial effects of TPE on clinical outcomes, but further data from randomized trials are expected. Until then, in my opinion, there is no role for TPE in non-severe HTG-AP. Insulin may be used in these cases, although it is also lacking evidence of efficacy and effect on clinical outcomes [43]. In severe HTG-AP, the question remains open, as other mechanisms (reduction of SIRS, elimination of FFA, etc.) may play a role in addition to triglyceride lowering. Furthermore, there is probably some heterogeneity among patients in the rate of triglyceride decline. Therefore, as previously suggested [73], TPE should be considered in those patients, in whom the decline within the first 12–24 h of conservative treatment (including insulin) is not as good as expected (e.g. 25% within 12 h or 50% within 24 h), especially if baseline triglycerides are very high and it would take several days for them to drop below 10 mmol/l. TPE should be considered in cases of HTG-AP occurring during pregnancy, as there are additional risks from the disease (premature labor, fetal death), while TPE is known to be a very safe treatment during pregnancy. Combined treatment with insulin and TPE is also a possibility. Further randomized trials are necessary to support the use of both, insulin therapy and TPE in HTG-AP. The results of the ELEPHANT trial [64], expected by 2025, will likely settle the role of TPE and insulin therapy in the near future.

## Data Availability

All data collected from the literature are available within the article.
